# Objective comparison of lesion detectability in low and medium-energy collimator iodine-123 mIBG images using a channelized Hotelling observer

**DOI:** 10.1088/1361-6560/62/1/17

**Published:** 2016-12-13

**Authors:** Rebecca A Gregory, Iain Murray, Jonathan Gear, Matthew D Aldridge, Daniel Levine, Lucy Fowkes, Wendy A Waddington, Sue Chua, Glenn Flux

**Affiliations:** 1 Department of Physics, the Royal Marsden NHS Foundation Trust and Institute of Cancer Research, Downs Road, Sutton SM2 5PT, UK; 2 UCL Institute of Nuclear Medicine and UCL Hospitals NHS Foundation Trust, 235 Euston Road, London NW1 2BU, UK; 3 Department of Nuclear Medicine and PET/CT, The Royal Marsden NHS Foundation Trust, Downs Road, Sutton SM2 5PT, UK; rebecca.gregory@icr.ac.uk

**Keywords:** iodine-123, neuroblastoma, collimator, channelized Hotelling observer, lesion detectability

## Abstract

Iodine-123 mIBG imaging is widely regarded as a gold standard for diagnostic studies of neuroblastoma and adult neuroendocrine cancer although the optimal collimator for tumour imaging remains undetermined. Low-energy (LE) high-resolution (HR) collimators provide superior spatial resolution. However due to septal penetration of high-energy photons these provide poorer contrast than medium-energy (ME) general-purpose (GP) collimators. LEGP collimators improve count sensitivity. The aim of this study was to objectively compare the lesion detection efficiency of each collimator to determine the optimal collimator for diagnostic imaging.

The septal penetration and sensitivity of each collimator was assessed. Planar images of the patient abdomen were simulated with static scans of a Liqui-Phil^™^ anthropomorphic phantom with lesion-shaped inserts, acquired with LE and ME collimators on 3 different manufacturers’ gamma camera systems (Skylight (Philips), Intevo (Siemens) and Discovery (GE)). Two-hundred normal and 200 single-lesion abnormal images were created for each collimator. A channelized Hotelling observer (CHO) was developed and validated to score the images for the likelihood of an abnormality. The areas under receiver-operator characteristic (ROC) curves, *Az*, created from the scores were used to quantify lesion detectability. The CHO ROC curves for the LEHR collimators were inferior to the GP curves for all cameras. The LEHR collimators resulted in statistically significantly smaller *Az*s (*p*  <  0.05), of on average 0.891  ±  0.004, than for the MEGP collimators, 0.933  ±  0.004. In conclusion, the reduced background provided by MEGP collimators improved ^123^I mIBG image lesion detectability over LEHR collimators that provided better spatial resolution.

## Introduction

Radionuclide imaging with Metaiodobenzylguanidine (mIBG) has a fundamental role in the diagnosis, staging and evaluation of treatment response in childhood neuroblastoma and adult neuroendocrine tumours (NETs) (Gelfand 1993, Shapiro *et al* 2001, Brisse *et al* 2011). Scintigraphy is routinely undertaken using ^123^I-mIBG in preference to ^131^I-mIBG, because better quality images can be obtained from 159 keV ^123^I gamma photons and twenty times more ^123^I activity can be administered for diagnostic scans (Shapiro *et al* 1987, Matthay *et al* 2010). Iodine-123 is also advocated for treatment planning prior to ^131^I mIBG therapy (Monsieurs *et al* 2002), despite the fact that the images obtained have been shown to be less sensitive in detecting lesions than post-therapy ^131^I-mIBG scans (Yang *et al* 2012). In response an alternative positron emission tomography tracer, ^124^I, has been used in attempts to improve diagnostic image quality and quantitative accuracy for dosimetry (Huang *et al* 2014, Koopmans *et al* 2014). As ^124^I-mIBG is not widely available and its clinical efficacy remains unproven, ^123^I-mIBG remains the standard.

**Figure 1.**
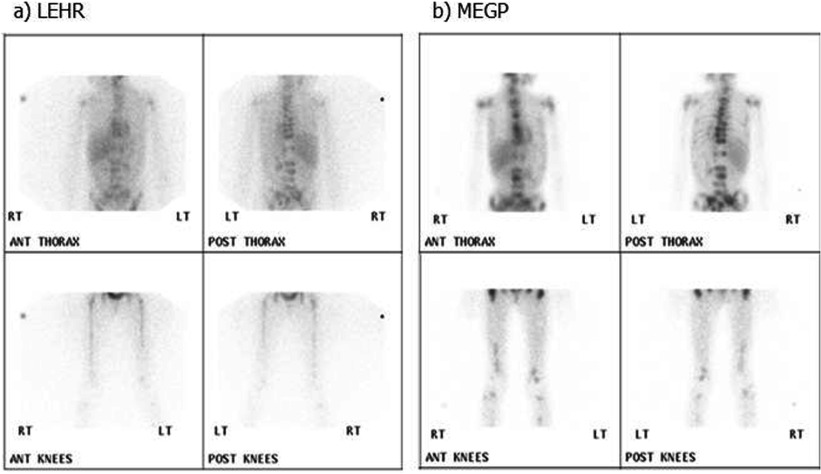
Static images of a 7 year old child with metastatic neuroblastoma deposits A acquired with a high-resolution collimator then B with a MEGP collimator.

Although ^123^I predominantly emits gamma photons at 159 keV (83% abundance), photons above 500 keV (2.3% abundance) are also emitted. These high energy photons penetrate the septa of low-energy (LE) collimators and down-scatter into the main photo peak energy window, producing background counts that reduce image contrast (Dobbeleir *et al* 1999). It was demonstrated almost 40 years ago that medium-energy general purpose (MEGP) collimators provide better signal detection than the a LE high-resolution (HR) collimators (Bolmsjo *et al* 1977). A number of groups now recommend the use of MEGP collimators for ^123^I-mIBG imaging based on a qualitative assessment (Macey *et al* 1986, Dobbeleir *et al* 1999, Snay *et al* 2011, Gelfand *et al* 2013). However the spatial resolution provided by a MEGP collimator is typically poorer than that provided by a LEHR collimator and its count sensitivity lower than its LE general-purpose (GP) counterpart. Consequently the choice of collimator may significantly impact upon image quality and ultimately interpretation of the ^123^I-mIBG study. Figure 1 demonstrates the difference in appearance of 10 min static scans for the same patients acquired with Philips’ high-resolution and MEGP collimators.

Whilst there are well established guidelines on the use of ^123^I-mIBG imaging in neuroblastoma and NET cases, explicit guidance with regard to collimator use is lacking. The choice of collimator is left to the discretion of the imaging centre (Olivier *et al* 2003, Bombardieri *et al* 2010, Matthay *et al* 2010, Taieb *et al* 2012).

The aim of this study was to determine which collimator optimises lesion detection on planar ^123^I mIBG images. The channelized Hotelling observer (CHO) model can be used to rapidly score larger sets of images than humans for the presence of an abnormality providing good statistical quality receiver operator characteristic (ROC) curves, that can be used to assess the efficacy of lesion detection for different imaging methods. This model has been shown to correlate well with human observers in lesion detection tasks and has been widely used in the optimization of several nuclear medicine imaging techniques (Hanley *et al* 1982, Gifford *et al* 2000, Gifford *et al* 2005, He *et al* 2010, El Fakhri *et al* 2011, Bal *et al* 2014, Yang *et al* 2014). In this study our implementation of the CHO model was first validated against human observations of 100 images for 2 collimators. The validated model was then applied to score 400 test images generated from 8 separate collimators from 3 different manufacturers. ROC curves were produced from these scores for each collimator. The areas under the ROC curves were used to provide an objective measure of the lesion detectability. These values were compared to objectively demonstrate the collimator that optimised lesion detection.

## Methods

Collimator design varies widely between manufacturers. Therefore collimators on a Discovery NMCT (GE), a Skylight gamma camera (Philips) and a Symbia Intevo Excel SPECT/CT (Siemens) were assessed (table 1). It should be noted that the Skylight camera had thicker crystals (5/8″) than the Discovery and Intevo (3/8″) models.

**Table 1.** Collimator characteristics with NEMA septal penetration and system sensitivity.

Camera	Collimator	Hole size^a^ (mm)	Septal thickness^a^ (mm)	Length (mm)	Spatial resolution (mm)^a^	Septal pen-etration %	System sensitivity (cps/MBq) ± error^b^
Discovery (GE)	MEGP	3.0	1.05	58.0	9.4	0.0	70.3 ± 1.2
	LEHR	1.50	0.20	35.0	7.4	0.2	96.6 ± 1.7

Skylight (Philips)	MEGP	2.95	1.14	48.0	11.3	0.0	101.1 ± 1.9
	LEHR	1.40	0.15	32.8	7.4	1.1	109.3 ± 2.1
	LEGP	1.40	0.18	24.7	8.8	5.8	204.3 ± 3.9

Intevo (Siemens)	MELP	2.94	1.14	40.64	12.5	0.0	115.6 ± 0.5
	LEHR	1.11	0.16	24.05	7.5	16.3	185.9 ± 0.9
	LEAP	1.45	0.2	24.05	9.4	12.0	225.9 ± 1.0

MEGP/LP  =  medium-energy general purpose/low penetration, LEG/AP  =  low-energy general/all purpose and LEHR  =  low-energy high-resolution.

a Spatial resolution is supplied for collimated photons, as the FWHM at 10 cm from the collimator external surface in the manufacturers specification, for a 3/8″ crystal. The thicker (5/8″) Skylight crystal will provide poorer spatial resolution, however this value is not available from the manufacturer.

b Error propagated from the uncertainty in the measured activity and square root of the region of interest counts, used in the NEMA calculation of sensitivity.

### Collimator characterisation

Each collimator’s septal penetration fraction and system sensitivity were measured for ^123^I imaging in accordance with the NEMA standards (NEMA 2007).

### Physiological background and lesion present patient image simulation with anthropomorphic phantoms

Images were created using an anthropomorphic phantom to simulate the clinical challenge of lesion detection for each collimator/camera combination.

**Figure 2.**
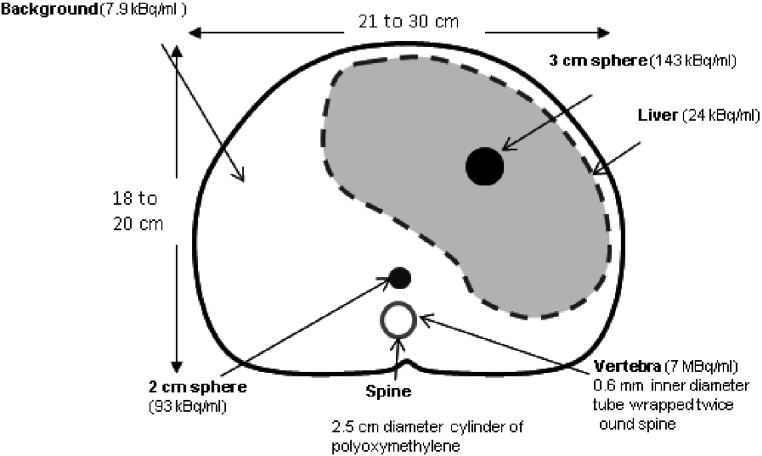
A cross-section of the abdomen-shaped phantom. The positions and activity concentrations of the fillable inserts are shown. These were scanned individually within the abdomen shaped shell alongside the spine insert. The shell dimensions vary within the range indicated along the axial length of the phantom.

The Liqui-Phil^™^ anthropomorphic abdomen-shaped phantom was filled with 9585 ml inactive water. The dimensions of this phantom, indicated in figure 2, correspond to an older child or adult. The phantom contained an inactive 2.5 cm diameter polyoxymethylene cylinder to simulate the attenuation of the spine. Lesion-mimicking-inserts filled with ^123^I were positioned in the phantom at the positions shown in figure 2. These were a 2 cm diameter sphere adjacent to the anterior surface of the spine, a 3 cm diameter sphere near the centre of the liver and (1 mm internal diameter) Polyethylene tubing wrapped twice around the spinal cylinder to represent a single mIBG avid vertebra. Three separate anterior and posterior pairs of planar scans were acquired with the abdomen-shaped phantom containing only one lesion at a time. In addition the Liqui-Phil^™^ liver insert filled with ^123^I was imaged separately within the abdomen-shaped phantom. Finally ^123^I was added to the abdomen-shaped phantom itself and it was imaged independently to produce an abdominal background image. The activity concentrations used for all inserts and the background are shown in figure 2. The phantom was positioned on the standard imaging couch. Planar scans were acquired with each collimator on each gamma camera with a 20% wide energy window centred on 159 keV, a 256  ×  256 matrix and uniformity correction. The uniformity correction on the Skylight was based on intrinsic ^123^I maps, to correct for photon detector tube edge enhancement artefacts. No such artefacts were observed in these phantom images. This was not necessary for the other 2 systems and an extrinsic Cobalt-57 uniformity map was used. The pixel dimensions were 4.6  ±  0.2 mm^2^ as the detector field-of-view varies for each camera. The Siemens Intevo has the largest 614 mm FOV, the Philips Skylight has a 597 mm FOV and the GE Discovery has the smallest 565 mm FOV. The collimator faces were positioned as close as possible to the phantom posterior and anterior surfaces. The objects were scanned for as long as possible depending on each camera’s availability. The frame duration used for each collimator image set was adjusted to account for the physical decay of the ^123^I between each image set, so that the image count statistics remained comparable. Therefore these high-count images reflect the relative variation in sensitivity, spatial resolution and septal penetration between the collimators. A minimum of 3k counts was collected for the inserts and 347k counts for the abdomen. For each image set acquired the abdomen-shaped phantom position was accurately reproduced using markings on the camera couch.

The acquired images were combined using an interactive data language (IDL) (release 8.2.3, ITT Visual Information Solutions) user script. This program first reduced the liver and abdomen image counts to values representing clinical image frame times between 400 and 600 s. The count in each pixel was then randomly selected from a Poisson distribution, with a mean value set to the rescaled count. A different random seed was used to create 200 different statistical realisations of the liver and abdomen. These images were then summed to produce 200 background images per collimator of the liver and physiological abdominal background images with liver-to-background ratios (LBR) ranging from 3.6 to 5.6.

For each collimator 200 images containing a low-contrast single-lesion were created by adding lesion-insert-images to the background images. The lesion-insert-image counts were rescaled prior to Poisson resampling to simulate varied tumour-to-background ratios (TBR). The tumour images were also shifted axially to simulate a variety of lesion locations. The resampled-shifted-lesion-insert images were then summed to the normal scans. In this way 17 images with the 3 cm sphere in the liver, 79 with the 2 cm sphere next to the spine and 104 images with a vertebra were created, all with different TBRs. The resulting liver-lesion TBRs ranged from 2.8 to 7.2, the spine-lesion TBRs were 29 to 255 and the vertebra TBRs were 179–1785. The vertebra required a high TBR to be visible. The range of simulated activity concentrations are shown in table 2. These lesion-to-background activity concentration ratios and positions were chosen to provide a broad range of challenging lesion detection tasks, from clearly visible to barely perceivable lesions.

**Table 2.** Simulated activity concentration ranges in the resampled phantom images.

Object	Simulated Range (kBq ml^−1^)
Abdomen	0.53–0.79
Liver	1.6–2.4
3 cm sphere in liver	36–143
2 cm sphere by spine	23–202
Vertebra	1000–7000

A total of 3200 posterior images were used for CHO scoring. The resultant average signal-to-noise ratios (SNR) in 3 cm circular ROIs in the hottest part of the liver and coldest part of the background were compared to those measured in 6 recent patient scans. Three of these 10 min static patient scans were acquired with MEGP collimators and 3 were acquired with LEHR collimators. The SNR was calculated as the mean divided by the standard deviation in the mean ROI counts.

### Channelized Hotelling observer

A mathematical model, the channelized Hotelling observer (CHO) was used to score the images for the possibility of an abnormality. The CHO model was written in IDL following the method published by Shidahara *et al* (2006). Test statistics were produced from the Fourier transformed images, **F(***ρ*), filtered into different frequency channels. These channels have a psychophysiological basis in reflecting the frequency selective channels of the human visual system. Radially symmetric channels were applied in the frequency domain, described by;

1 \begin{eqnarray*}{{u}_{\text{c}}}\left(\rho \right)=\left\{\begin{array}{c} 1\parallel \rho \parallel \in \left[{{\rho}_{0}}{{2}^{c-1}},{{\rho}_{0}}{{2}^{c}}\right] \\ 0~\,\text{Otherwise} \end{array},\right. \end{eqnarray*}

where $c\in \left\{1,2,3,4,5\right\}$ ${{\rho}_{0}}$ pixel^−1^ is the low end cut-off frequency. The average value in each channel was calculated to create a matrix of values, *r*_*i*_, for each of the 5 channels, *i*, **r**  =  [*r*_1_, *r*_2_, *r*_3_, *r*_4_, *r*_5_] for each image *f*. The difference of the means of the channel matrices per camera-collimator combination, for the lesion present ${\boldsymbol{\overline{r}}_{a}}$ and lesion absent ${\boldsymbol{\overline{r}}_{n}}$ channel matrices were used to define a matching filter ${\boldsymbol{\overline{r}}_{a}}-{\boldsymbol{\overline{r}}_{n}}$.

Pre-whitening was used to de-correlate the noise in the channels by multiplication with ${{\mathbf{K}}^{-1}}$, which is the inverse of the average of the 2 noise covariance matrices for the entire abnormal and normal ensemble. Then a test statistic *λ*_*f*_ for each image was calculated as;

2 \begin{eqnarray*}{{\lambda}_{f}}={{\left[{\boldsymbol{\overline{r}}_{a}}-{\boldsymbol{\overline{r}}_{n}}\right]}^{T}}{{\mathbf{K}}^{-1}}\mathbf{r}.\end{eqnarray*}

In effect CHO provides a score for the possibility of a lesion presence, as the weighted sum of the counts in the frequency channels of the tested image.

### Channelized Hotelling observer validation

In order to validate the CHO model, 4 experienced human observers scored 240 of the posterior phantom images for the presence of a lesion, using the scoring system in table 3. The observers were 2 medical physicists and 2 radiologists all with over 10 years’ experience at interpreting nuclear medicine scans. The images consisted of 60 of the background and 60 images containing lesion inserts generated for each of the Philips LEHR and MEGP collimators. These were viewed on the Hermes work station (Hermes Medical Solutions) that all the observers were familiar with. The images from the different collimators were randomly interspersed so the observers were not informed of the collimator used. The first 20 images from each collimator were used as training sets as the observer learnt the scoring system and adjusted their own criteria to apply it. The following 100 images were used to form ROC curves. For each score for each of the background and lesion present images, the fraction of the scores equal to and above a threshold score was calculated. The threshold that was used to assign an image as positive or negative for the presence of a lesion, was incremented from the lowest to the highest score (1–5). The true positive fraction (TPF) was calculated from the lesion present images and the false positive fraction (FPF) was calculated from the background images. The average TPF and FPF for each human score threshold was used to create an average human ROC curve against which the CHO curves were compared.

**Table 3.** Human observer scoring system.

Score	Decision
1	Definitely normal
2	Probably normal
3	Equivocal
4	Maybe abnormal
5	Definitely ab normal

CHO was used to score the same 100 (non-training) images for each collimator. CHO scores were binned into 12 score thresholds equally covering the range of scores generated by CHO for each data set. This gave comparable discrete FPF sampling frequency for the CHO and human ROC curves for validation. The area under these ROC curves, *Az*, was then calculated using a trapezoidal method. Standard deviations on the data points were calculated according to Metz (Metz 1978). These standard deviations were propagated to estimate errors on the area under the ROC curves. The CHO cut-off frequency was adjusted to achieve the best agreement between the CHO and average human observer *Az*. The validated cut-off frequency was used for the further comparison of collimators.

### Collimator lesion detectability comparison

The full set of 3200 posterior anthropomorphic phantom images was scored using the validated CHO model with the optimised cut-off frequency. The anterior images were discarded as neither observer was able to identify the lesions in the images leading to ineffectual ROC curves with an *Az* of 0.5.

The 400 CHO scores for each collimator were used to generate ROC curves for near continuous thresholds using ROCkit 0.9B (Metz 1998). This software also fitted a binormal distribution to the ROC curve, calculated *Az*, compared the correlated ROC curves and provided a two-tailed p-value to quantify the statistical significance of the difference between them. It had not been possible to apply this software to the human scores due to limited number of data points associated with the 5 human scores.

## Results

### Collimator characterisation

Table 1 shows the NEMA test results alongside the other collimator properties. No septal penetration was found for any of the medium-energy collimators. The long thick septa of these collimators appeared to stop the high energy photons. However the ME collimators were the least sensitive for each camera. The LEGP collimators were the most sensitive and gave double the sensitivity of the MEGP collimators but with over 5% septal penetration. The most septal penetration (16.3%) occurred for the Siemens LEHR collimator which has the shortest thin septa of all the collimators tested. Star artefacts were evident in the images from this collimator. The septal penetration of the Siemens’ LEHR collimator was 15 times higher than that of the Philips, due to the shorter Siemens’ collimator holes. The GE collimators provided the lowest septal penetration of the three manufacturers.

The sensitivity between camera collimators could not be compared because the thicker 5/8″ crystal of the Skylight (Philips) will bias these sensitivity results.

### Physiological Background and Lesion Present Patient Image Simulation with Anthropomorphic Phantoms

**Figure 3.**
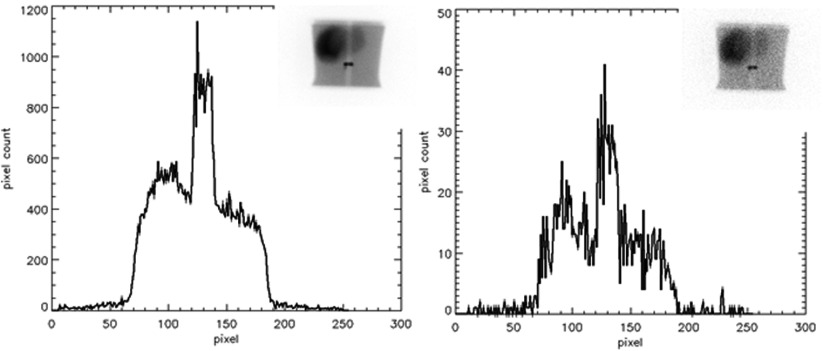
An example of a high count image prior to Poisson resampling left, with a profile through the insert image overlaying the spine to mimic one active vetebra. The image on the right is the same geometry following Poisson resampling.

Images before and after Poisson resampling are shown in figure 3. These are inset on a plot of a single pixel wide profile across the vertebra insert.

**Figure 4.**
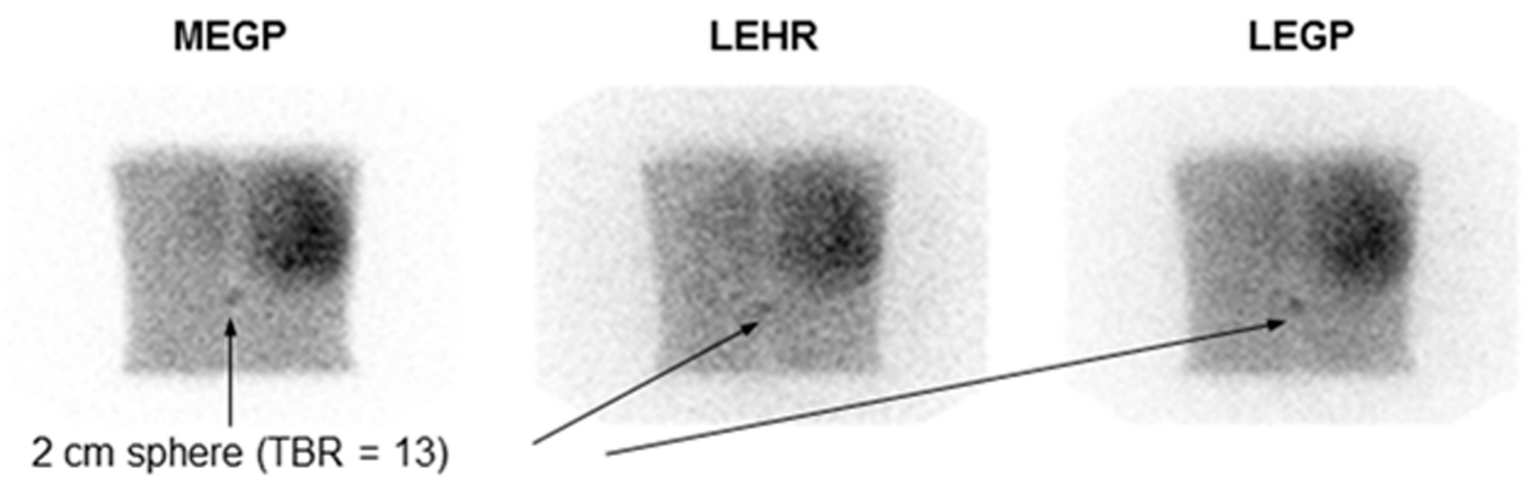
Example abnormal posterior images from the Skylight (Philips) gamma camera, corresponding to each of the available collimators.

The resultant simulated total image counts ranged from 245k to 378k counts, which are typical of those acquired from 10 min abdominal mIBG planar scans. Examples of the generated images are shown in figure 4. The average pixel count in the background of the 6 patient scans was 20  ±  5 compared to 15  ±  5 in the background of the phantom scans. The average liver counts were 45  ±  7 and 30  ±  8 in the patient and phantom images respectively. The average liver-to-background ratio (LBR) counts were 2.26  ±  0.89 and 1.96  ±  1.59 in the patient and phantom images respectively. The SNR in the patient images background was 4.16  ±  0.59 and the liver was 6.49  ±  1.38. In the phantom images the SNRs were 3.47  ±  0.36 in the background and 4.73  ±  0.44 in the liver. All errors on these values are the standard deviations in the means. The count densities and SNRs were therefore lower in the phantom than the patient images, but a similar order of magnitude.

### Channelized Hotelling observer validation

**Figure 5.**
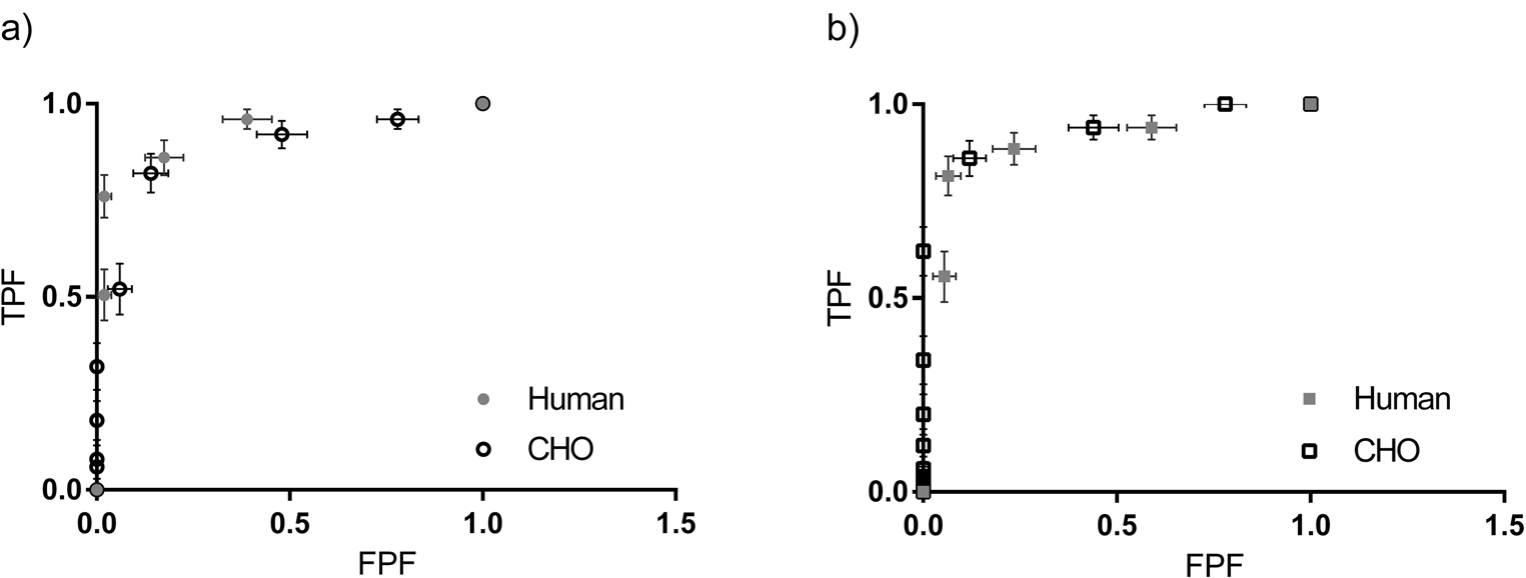
CHO with a 0.008/cycle cut-off frequency versus the average human ROC curves for (a) the LEHR and (b) MEGP posterior images generated for the Philips Skylight gamma camera. The errors on the human curves are the standard deviation in the FPFs and TPFs.

The least difference between the human observer and CHO ROC *Az* scores was observed at a low end cut-off frequency of 0.008 pixel^−1^. This was the lowest achievable frequency at 2 per 256 pixels. This value of ${{\rho}_{0}}$ was used for all subsequent CHO experiments. The validation ROC curves are shown in figure 5, the areas under these ROC curves are shown in table 4. The values are within the standard errors of one another.

**Table 4.** CHO Validation (120 images for each collimator): areas under the ROC curves (figure 5) *Az*  ±  standard error.

Collimator	Human	CHO
MEGP	0.89 ± 0.11	0.93 ± 0.12
LEHR	0.92 ± 0.11	0.87 ± 0.12

### Collimator lesion detectability comparison using receiver operator characteristic curves

**Figure 6.**
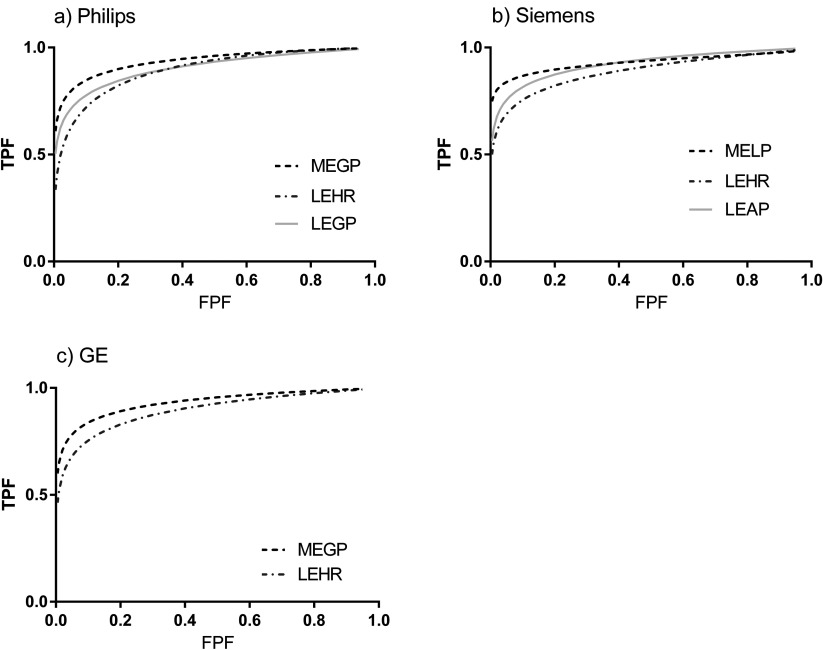
The binormal fit to the ROC curves for each manufacturer and tested collimator.

The ROC curves generated using CHO from 200 normal and 200 abnormal images for each collimator are shown in figure 6. The LEHR ROC curves consistently demonstrated lower TPF values than for the GP curves indicating poorer lesion detectability with LEHR collimators than for the other collimators.

The areas under these curves are given in table 5. On average the LEHR collimators resulted in smaller *Az*s of 0.891  ±  0.004 than 0.933  ±  0.004 for the MEGP collimators. This difference was statistically significant (*p*  <  0.05) see table 6. There was no statistically significant difference between the Philips LEGP and LEHR collimators. However there was a statistical difference between the *Az* for the equivalent Siemens collimators. In contrast there was a statistically significant difference between the Philips MEGP and LEGP collimators but not for the corresponding Siemens collimators.

**Table 5.** CHO collimator comparison (400 images for each collimator): areas under the ROC curves (figure 6) *Az*  ±  standard error.

Manufacturer	Collimator	*Az*
GE	MEGP	0.932 ± 0.013
	LEHR	0.894 ± 0.016

Philips	MEGP	0.937 ± 0.012
	LEHR	0.893 ± 0.016
	LEGP	0.903 ± 0.016

Siemens	MELP	0.929 ± 0.015
	LEHR	0.887 ± 0.017
	LEAP	0.920 ± 0.014

**Table 6.** Two tailed *p*-values of correlation between collimators *Az*.

Manufacturer	Collimators	*p*-value
GE	MEGP versus LEHR	0.029

Philips	MEGP versus LEHR	0.012
	LEHR versus LEGP	0.657
	MEGP versus LEGP	0.039

Siemens	MELP versus LEHR	0.005
	LEHR versus LEAP	0.011
	MELP versus LEAP	0.563

## Discussion

Eight collimators from the three major manufacturers were characterised and assessed for ^123^I imaging in this study. These cover the majority of the collimators currently used for ^123^I imaging. Collimators designed specifically for ^123^I imaging are not in wide use and were not available for this study.

Septal penetration was measured for all the low-energy collimators. The Siemens LEHR collimators gave the highest levels of 16.3% septal penetration. At acceptance testing of the Intevo system (Siemens) it was noted that this collimator similarly suffered from high (9.4%) septal penetration with Tc-99m. This may be expected as the Siemens LEHR collimators have the smallest holes and shortest thin septa of the collimators tested.

The MEGP collimators allowed no measurable septal penetration, therefore the count rates did not vary with source-collimator distance. Future work will investigate the optimal protocol for quantitative imaging to provide standard uptake values for early assessment of treatment response and dosimetry calculations. Quantification should be simpler using MEGP collimators as the counts did not vary with source-collimator distance. The count rates for the low-energy collimators did vary with this distance due to septal penetration, so the quantitative accuracy for these images would vary with the activity distribution. Therefore benefits from using MEGP over low-energy collimators for quantitative imaging are expected and have been demonstrated for cardiac imaging (Inoue *et al* 2003).

The areas under the CHO and human ROC curves agree within the standard errors of each other. Therefore the CHO model was successfully validated against human scoring (figure 5 and table 4). The smallest possible low end cut-off frequency (0.008 pixels^−1^) for a 256 matrix gave the best agreement between the CHO and human observer areas under the ROC curves, *Az*. This corresponds to scoring images at the lowest usable 5 frequency bands, where statistical noise is effectively filtered. Therefore the variations in count concentrations correspond to structural rather than statistical changes. The human observer would also concentrate on these lower frequencies to identify structures, such as lesions. Therefore this finding is as expected.

The areas under each of the collimator’s human ROC curves were similar at 0.92  ±  0.11 and 0.89  ±  0.11 for the MEGP and LEHR collimators respectively. However ROCkit could not be used to fit a ROC curve to the human’s scores, due the limited number of data points. The ROC curve trapezoidal *Az* was therefore calculated manually. These curves were not compared to assess the statistical significance of the difference in *Az* between the collimators from human scores. However the overlapping error intervals on the areas under the ROC curves in table 4 indicate the difference in *Az* between collimators is insignificant.

The ROC curves for CHO achieved from large image sets (200) of images per collimator provided improved statistical quality ROC curves compared to that attained with an ensemble of 50 images and human scores. The low errors on the *Az* from these ROC curves allow demonstration of the significant differences in *Az* for some of the collimators with confidence in 2-tailed *p*-values less than 0.05 (table 6). The variations in collimator and couch designs and pixel size between the manufacture’s systems led to a 5% range in *Az*, of 0.05 compared to the average *Az* of 0.91.

CHO scores are based on the counts within the low frequency channels. The CHO ROC curves obtained from the MEGP collimators were consistently highest due to the larger number of low frequency counts in the MEGP images corresponding to lesions (figure 6). Septal penetration causes a higher proportion of counts from the lesions to appear at higher frequencies within the LEHR collimator images, which additionally increases the image noise and reduces image contrast. Therefore the areas under the ROC curves (table 5) objectively demonstrate improved lesion detectability with MEGP compared to LEHR collimators, due to these factors. These results are in agreement with published findings that MEGP collimators demonstrate improved ^123^I image quality (Bolmsjo *et al* 1977, Snay *et al* 2011, Gelfand *et al* 2013). The improvement in lesion visibility with general purpose collimators is also evident in the example images of figure 3.

A limitation of this work was that test data were necessarily generated from phantom images, as it is impractical to acquire 200 patient images of normal physiology and 200 images with a single lesion on multiple systems. The solution in this study was to simulate these using Poisson resampled phantom images. The generated images in figure 3 have similar appearance to the abdominal region of figure 1. Although the count densities and SNRs are lower in the phantom-based simulations than in the average patient images, they are the right order of magnitude and therefore realistic. The liver-to-background ratios were varied to simulate the physiological differences in patients and were on average similar to those measured in a sample of clinical scans. However using the phantoms available for this study it was not possible to simulate variations in patient geometries. Therefore the training images were similar to those used to produce the ROC curves.

This assessment was performed for static scans as lesions tend to be identified on planar images and these images may be used for disease staging alone. If SPECT images are used the lesions are first located in the planar scans to identify the region where the SPECT scan is performed. These 3D images are then generally used to confirm the lesion location after it has been detected (Barwick *et al* 2010). This work may be extended to optimise the many acquisition and reconstruction parameters for SPECT imaging to improve lesion detection and therefore localisation.

## Conclusions

In this study a channelized Hotelling observer model was developed, validated and used to objectively quantify lesion detectability as the area under ROC curves. This demonstrated that the benefit of reduced background noise, provided by the general-purpose collimators, outweighs the benefit of the superior spatial resolution of LEHR collimators for mIBG avid lesion detection using ^123^I mIBG planar images.
